# To Say or Not to Say: Medical and Social Project

**DOI:** 10.17650/2712-7672-2020-1-2-72-76

**Published:** 2020-12-04

**Authors:** Liana N. Abramova, Ekaterina V. Shakhova

**Affiliations:** Mental-health clinic No. 1 named after N.A. Alexeev

**Keywords:** education, stigma, prevention of social stigma, mental disorder, psychiatry, просвещение, стигма, дестигматизация, психическое расстройство, психиатрия

## Abstract

Mental health and psychological education activities are being carried out in Moscow (the Russian Federation), along with measures aimed at prevention of social stigma in mental health care. The medical, social and educational project *To Say or Not to Say* has been developed by a group of experts from the Mental-health clinic No. 1 named after N.A. Alexeev, for Moscow residents. The title chosen for the project urges participants to make a choice: continue living with their problems or take a step towards solving them. A new educational activity format has been created and tested in the course of the project, and this format provides an opportunity to largely overcome the stigmatizing barriers that prevent people from seeking psychological and psychiatric help in Moscow. Sixteen events involving over 7,000 citizens have been held, and the psychiatrists engaged in the project have spent 2,280 man-hours in this volunteering activity. We believe that this educational activity could help to overcome social stigma in psychiatry, further research is needed to measure the effect of our educational project on social stigma associated with mental health.

Tolerance, declared to be a civil society standard in the Declaration of Principles on Tolerance at the UNESCO General Conference on 16 November 1955, has since attained even higher status and is now seen as a moral imperative. This is a widely accepted fact. Generally, the term tolerance means tolerance of any other view of the world, lifestyle, behaviour and customs. Furthermore, it is important to understand that tolerance is not equal to indifference.

People with mental and behavioural disorders often experience social stigma. According to the World Health Organization, breach of freedoms and limitation of civil, political, economic, social and cultural rights for people with mental disorders are typical of many countries and take place both inside and outside healthcare facilities.

Social stigma is distressing not only for patients but also their families. Mental illness in a family member often causes alienation from other family members, which is why families may attempt to hide this fact, thus creating barriers between the person who is sick and access to modern professional medical assistance. People who need psychiatric care do not seek doctors' assistance promptly due to psychological patterns and social stereotypes. This causes further emotional stress in addition to that caused by the mental illness itself, which makes individuals feel different from other people, and worsens social adaptation by preventing sufferers from living a normal social life. It also makes them feel guilty. Such people often experience a sense of social stigma that is directed towards themselves, with different reactions to the disease being internalized.

A more tolerant attitude towards people with mental disorders may be created, firstly, by means of prevention of social stigma - by various actions aimed at gradually easing off stigmatizing perceptions of a sick person. It is therefore important to inform people about any social prejudice and help society understand the psychological nature of people and the risk factors that may cause mental diseases.

The Department of Health, Department of Labour and Social Protection, and the Ministry of Civil Defence, Emergencies and Disaster Relief of the Russian Federation are now working in Moscow (Russia) on prevention of mental and behavioural disorders, and development of a behavioural health system. The Moscow Psychological Counselling Service has been created with a 24/7 psychological support hotline. The centre for emergency psychological assistance of the Russian EMERCOM renders psychological assistance to people who have suffered in emergency situations and to people who have found themselves in complicated conditions. Moscow's psychiatric service organizes different events, conferences and forums, including those on prevention and public education.

Taking into account the need for real progress in mental health and psychological education, and measures to prevent social stigma in psychiatry, the group of experts of the Mental-health clinic No. 1 named after N.A. Alexeev, led by Chief Psychiatrist of the city of Moscow, Professor George P. Kostyuk, has developed the educational, medical and social project To Say or Not to Say, for the city. This phrase urges participants to make a choice: continue living with their problems or take a step towards solving them.

**Figure 1:**
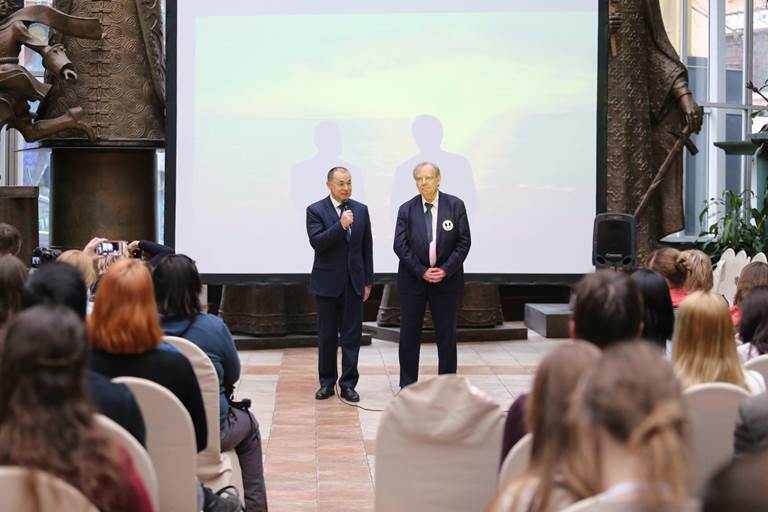
Moscow’s Chief Psychiatrist, Prof. George P. Kostyuk (on the left), and President of the Interregional Non-Governmental Organization of the Psychiatrists’ Club, Arkadij L. Shmilovich (on the right), at the event related to the To Say or Not to Say project, in Zurab Tsereteli’s gallery (22 November 2018). Photo by Aleksandr Iu. Shchapin.

**Figure 2:**
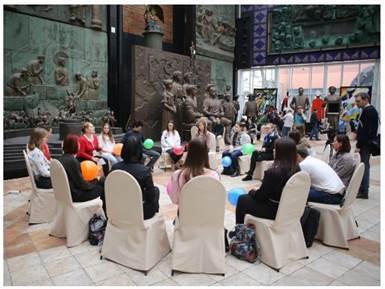
Group psychological training, part of the To Say or Not to Say project in Zurab Tsereteli’s gallery (22 November 2018). Photo by Aleksandr Iu. Shchapin.

Considering aspects of such a complicated subject as stigmatization of patients with mental disorders in society, the team has made the project modern and unique. It is far removed from classical ideas of psychiatry, mental disorders and defects in people's mental health. The event is for the general public: young people and students, middle-aged and elderly people, families with children, family members and close friends of people with mental disabilities.

**Figure 3:**
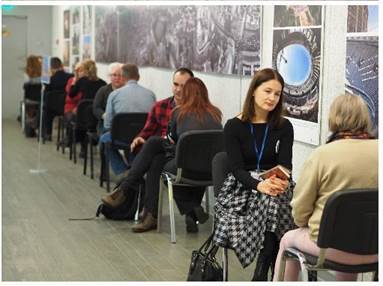
Individual counselling, part of the To Say or Not to Say project in Zurab Tsereteli’s gallery (22 November 2018). Photo by Aleksandr Iu. Shchapin.

**Figure 4:**
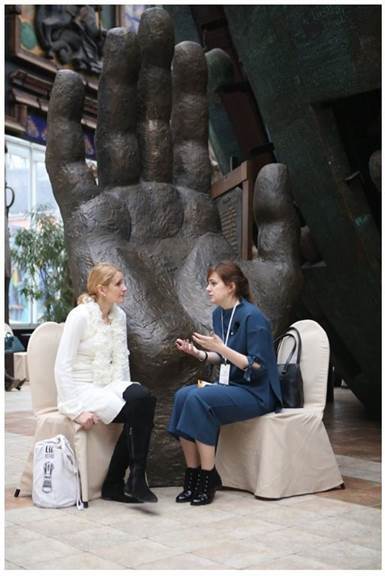
Individual counselling, part of the To Say or Not to Say project in Dom na Brestskoy (13 October 2018). Photo by Aleksandr Iu. Shchapin.

The idea of "open psychiatry" was used as a foundation for the project's communication strategy, as society generally sees psychiatry as a closed, rather negative medical domain with lots of myths. To disprove these notions, it was decided to go beyond social stereotypes. Creation of a sustainable system of efficient dialogue between healthcare professionals and people outside hospitals became an organizational component of the strategy.

As the To Say or Not to Say project is particularly complex, each meeting lasts for five-six hours. The programme includes medical and therapeutic interventions (psychological and psychiatric consultancy support; psychodiagnostics and testing; art therapy workshops), information and education (science education lectures on psychiatry, online educational webinars, etc.), and creative components (musical, poetic and theatrical sketches performed by inclusive teams, and exhibitions of work created by artists who have experience of psychiatric disorders etc.).

A unique feature of the project is its informal and trusting atmosphere, which helps participants understand that they are not alone in their need for such assistance, support, relief and removal of barriers. The core of each campaign (and a key part of the project) is a lecture about mental health, which is, at the same time, the subject of the meeting. The meeting programme was devised to ensure that each participant can take part in most of the activities, depending on his or her interests. Voluntary consultants, workshop speakers and lecturers include employees of all specialized health clinics providing psychiatric assistance: psychiatrists, psychotherapists, clinical psychologists, social work specialists and administrative staff.

**Figure 5:**
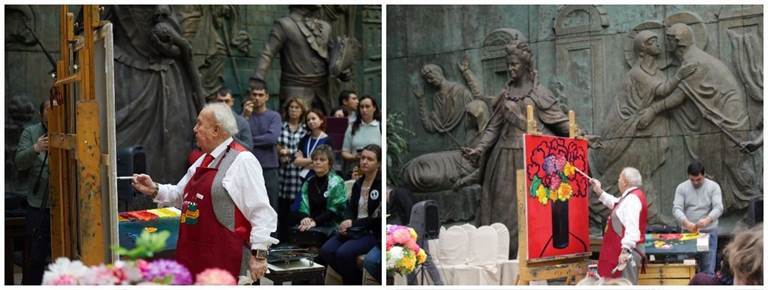
Workshop by renowned caricaturist, artist, essayist and former psychiatrist Andrei Biljo. To Say or Not to Say project's event in the Blagosfera, community center and creative spase (27 January 2019). Photo by Aleksandr Iu. Shchapin.

**Figure 6:**
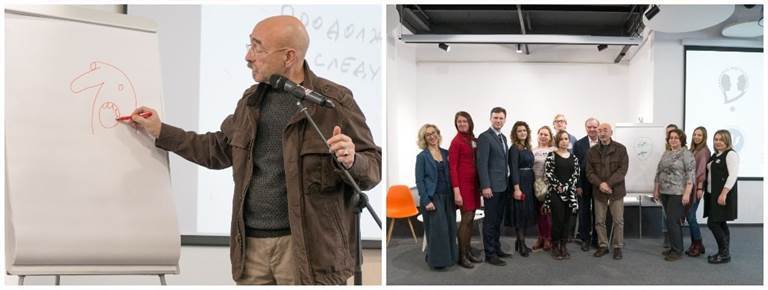
Workshop by Russian artist and sculptor Zurab Tsereteli, part of the To Say or Not to Say project in Zurab Tsereteli’s gallery (22 November 2018). Photo by Aleksandr Iu. Shchapin.

Workshop by renowned caricaturist, artist, essayist and former psychiatrist Andrei Biljo. To Say or Not to Say project's event in the Blagosfera, community center and creative spase (27 January 2019). Photo by Aleksandr Iu. Shchapin.

Workshop by Russian artist and sculptor Zurab Tsereteli, part of the To Say or Not to Say project in Zurab Tsereteli's gallery (22 November 2018). Photo by Aleksandr Iu. Shchapin.

The project has been functioning since 2018; offline educational campaigns (not internet-based) are held for Muscovites on a monthly basis, on a weekend day, in public and cultural zones of Moscow, during which participants can access the full range of expertise offered by Moscow's psychiatric service. In total, there were nine campaigns within the project for Muscovites in 2018 and seven in 2019. The total number of project participants during this two-year period was 7,000.

Most of the participants during all periods of the project were young people aged 19-25, i.e., people whom the team primarily focused on. The event was also popular among participants from other age groups, which, of course, proves its universal nature.

It is interesting that the largest number of attendants of all ages was in October and November 2018-2019. During these months, the project was implemented as a large-scale, city-wide campaign, and it coincided with both World Mental Health Day and Psychology Day. This has also contributed to its popularization and the active involvement of new participants.

Community organizations, mass media and social activists also joined the project.

By providing Muscovites with a positive experience of complex educational psychological work, the project has demonstrated its social significance and efficiency, covering a vulnerable social group - people with mental disorders and borderline states who feel psychologically uncomfortable and require consultations but are not yet ready to seek psychological and psychiatric assistance in healthcare institutions.

The following tasks have been carried out as part of the work performed:

1. The public's attention has been drawn to the need for discussion of people's mental health, considering the continual changes associated with the pace of life in modern societies and the appearance of new stress factors;
2. A trust-based dialogue platform has been created, especially for young people, addressing the matter of mental health, and measures have been put in place to overcome the barrier between users of psychological and psychiatric assistance and the specialists who provide such assistance;
3. A trust-based attitude to psychotherapists, psychiatrists and psychologists has been formed by providing regular, specialized advisory assistance and a mutual search for solutions and ways to solve problems;
4. Understanding of the lives of people who have experienced psychiatric problems has been expanded by demonstrating their creative abilities;

Different media have been incorporated into the project (to varying degrees), with a focus on creation and elaboration of a modern language for the media environment in this area and a respectful attitude towards psychiatry.

The project is currently active, and due to the COVID-19 pandemic, the main psychological and educational efforts are being implemented online.

## Acknowledgments

The authors would like to thank the following, who participated in the organization and holding of events in the To Say or Not to Say project: Chief Psychiatrist of Moscow, George P. Kostyuk; President of the Interregional Non-Governmental Organization of the Psychiatrists' Club (Psychiatry: Ariadne's Thread), Arkadij. L. Shmilovich; Deputy Head of the Medical Statistics Office of the Mental-health clinic No. 1 named after N.A. Alexeev, Aleksandr A. Avramenko; Social Work Specialist from the day hospital facility Il'mira Sh. Mansurova; and all the specialists and volunteers who contributed to the programme.

## Conflict of interest

The authors declare no conflict of interest.

## Funding

The authors declare that there was no funding for this work.

